# Benzoyl Peroxide's Sensitisation Potential and Potency in Experimental Methods and Review of Contact Allergy and Allergic Contact Dermatitis

**DOI:** 10.1111/cod.14765

**Published:** 2025-02-05

**Authors:** Kerstin Egele, Hans Drexler, Manigé Fartasch, Vera van Kampen, Hans F. Merk, Dennis Nowak, Axel Schnuch, Wolfgang Uter, Patricia Kreis, Brunhilde Blömeke

**Affiliations:** ^1^ Department of Environmental Toxicology Trier University Trier Germany; ^2^ Institute and Outpatient Clinic of Occupational, Social and Environmental Medicine Friedrich‐Alexander‐University Erlangen‐Nürnberg Erlangen Germany; ^3^ Dermatological & Allergological Private Practice Siegburg Germany; ^4^ Institute for Prevention and Occupational Medicine of the German Social Accident Insurance Institute of the Ruhr University Bochum (IPA) Bochum Germany; ^5^ Department of Dermatology and Allergology, Medical Faculty RWTH Aachen University Aachen Germany; ^6^ Institute and Outpatient Clinic for Occupational, Social and Environmental Medicine, Member of German Center for Lung Research (DZL), Comprehensive Pneumology Center (CPC München) Clinical Center of the Ludwig Maximilian University Munich (LMU) Munich Germany; ^7^ University Medical Center Göttingen Göttingen Germany; ^8^ Department of Medical Informatics, Biometry and Epidemiology (IMBE) Friedrich‐Alexander‐University Erlangen‐Nürnberg Erlangen Germany; ^9^ Department of Food Chemistry and Toxicology, Institute for Applied Biosciences Karlsruhe Institute of Technology (KIT) Karlsruhe Germany

**Keywords:** allergic contact dermatitis, benzoyl peroxide [CAS‐no. 94‐36‐0], non‐animal methods, occupational allergen

## Abstract

Positive patch test responses to benzoyl peroxide (BPO) have been reported from patients without and with known exposure. Up to 6.5% were found in the United States and 7.8% in a study including patients from Germany, Austria and Switzerland. We provide an overview of the skin sensitisation potential and potency of BPO based on animal experiments and non‐animal methods. BPO tested positive in Guinea Pig Tests and the Local Lymph Node Assay. Application of the current OECD guideline to identify a skin sensitizer by combining non‐animal method results gave differing outcomes. Moreover, patch test responses of patients to BPO were considered to determine the importance of BPO as a relevant occupational contact allergen. Another well‐known BPO exposure is the topical application to treat acne. Despite widespread use, extensive and long‐term skin exposure, we found for this group only studies reporting few positive patch test reactions. Further, occupational handling of BPO and contact allergy is reported by dental technicians. In‐depth evaluation of the prevalence of contact allergy in different professions with suspected BPO exposure did not reveal an association with occupational handling of BPO. Consequently, a generally increased risk for those professions is not supported.

## 1 Introduction

**FIGURE 1:**
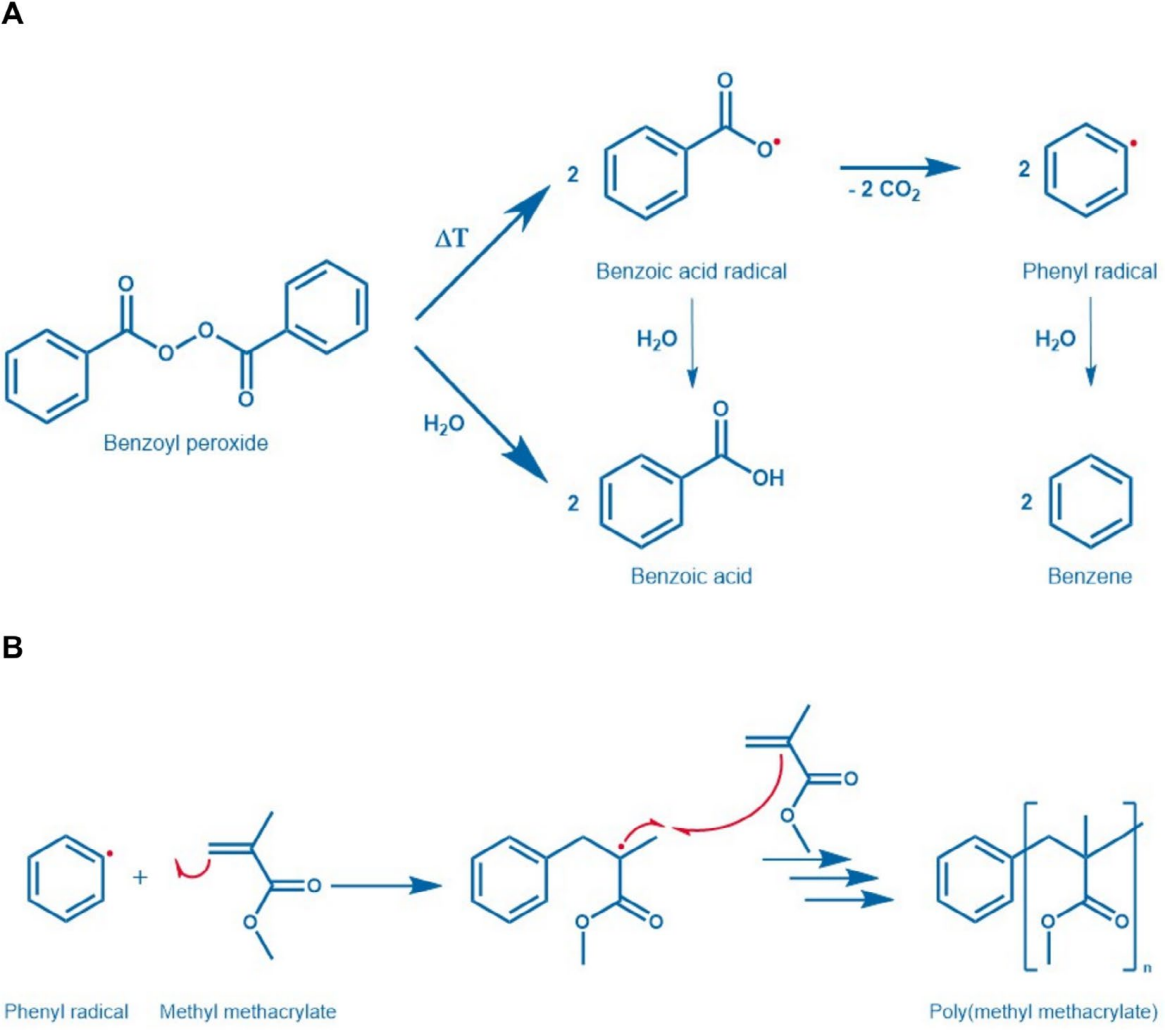
Chemical structure of BPO and mechanism of radical formation (A) and its use in free radical polymerisation of methyl methacrylate to poly methyl methacrylate (B).

Benzoyl peroxide (BPO, CAS‐no. 94‐36‐0) is used to manufacture a broad range of products. The chemical structure of BPO and the formation of benzoic acid, phenyl radical and benzene are shown Figure 1A. Under aqueous conditions BPO hydrolyses to benzoic acid/benzoate, while the formation of a stable phenyl radical by homolytic cleavage of the peroxide group is promoted by increased temperature. The BPO‐mediated methacrylate polymerisation reaction is shown in Figure 1B. BPO (~1 wt%) is a prominent initiator for radical polymerisations in the manufacturing of synthetic resins, plastics, adhesives, dental materials and bone cement [1, 2, 3, 4]. Furthermore, BPO (1%) was used to bleach candles and remains used in some countries to bleach flour and dairy products (lower ppm range) because of its oxidative properties [5, 6, 7].

The potential of BPO to induce skin sensitisation has been studied in experimental animal studies and non‐animal methods. BPO was tested in guinea pig tests such as the Guinea Pig Maximisation Test, Bühler Test and the murine Local Lymph Node Assay (LLNA) [8, 9, 10]. There is a common agreement that as of today, one single non‐animal test method is not sufficient to fully recapitulate the complexity of skin sensitisation. Thus, non‐animal methods are being developed along four defined key events (KE 1–4) of a defined Adverse Outcome Pathway for Skin Sensitisation (AOP) [11]. The first key event (KE 1) is the binding of electrophilic chemicals to nucleophilic centres of skin proteins, and OECD‐validated test methods (OECD 422 C) evaluate the relative reactivity of substances with synthetic model peptides containing cysteine and/or lysine (Direct Peptide Reactivity Assay, DPRA; kinetic DPRA, kDPRA). Other methods are using single amino acid derivatives instead of model peptides (Amino Acid Derivative Reactivity Assay, ADRA) [12]. Methods addressing the activation of keratinocytes (KE 2, OECD 442 D) are measuring the chemical‐induced changes in the expression of a marker gene in genetically modified HaCaT keratinocytes (KeratinoSens, LuSens) or gene expression of markers in human reconstructed epidermis (EpiSensA) [13]. Maturation of dendritic cells (KE 3, OECD 442 E) is evaluated by measuring cell surface expression of specific markers on monocytic THP‐1 or U937 cell lines (Human Cell Line Activation Test, h‐CLAT; U937 cell line activation Test, U‐SENS), cytokines (Interleukin‐8 Reporter Gene Assay, IL‐8 Luc assay) or expression of a set of marker genes (GARDskin) [14]. Validated methods to study the potential of a chemical to activate naive T cells (KE 4) are still under development. For the other methods validated test protocols are already provided by the Organisation for Economic Co‐operation and Development (OECD, guideline 442 C, D, E) [12, 13, 14]. Furthermore strategies (so‐called Defined Approaches) to combine individual results of testing KE 1–3, as well as in silico data of a given chemical into a final outcome was developed (OECD 497) [15]. BPO has been tested in several of the developed methods.

Humans may have skin contact under certain occupational settings, that is, during the synthesis of the molecule and manufacturing of products, while non‐occupational contact may be because of minimal residues of non‐reacted BPO during handling of BPO‐containing products [4, 16]. Consequently, under circumstances of intensive handling activities or non‐compliance with safety instructions of BPO‐containing products, skin sensitisation to BPO may occur. Independently, BPO itself is used in significant amounts in some drugs. In the past, BPO (< 20%) was used to treat leg ulcers in chronic venous insufficiency [17]. Up to 10% BPO has been used since the early 20th century and is considered a staple in treating acne, where high daily doses for several months are applied [18]. In the context of medical use and occupational handling cases of ACD have been reported [5, 17, 19].

The present article aims at reviewing available results of experimental animal studies, non‐animal methods and human patch test data to conclude on the human sensitisation potency of BPO and to identify occupational groups at risk for skin sensitisation.

## 2 Methodology of Literature Search and Data Analysis

Published data were collected (PubMed, Web of Science libraries). We included publications written in English and German Language. A systematic analysis of the literature of experimental animal studies and non‐animal methods were performed using the following keywords ((BPO) OR 94–36‐0) AND ((guinea pig maximisation test) OR (Bühler test) OR Buehler OR LLNA OR GPMT OR LLNA) or ((Peptide reactivity) OR (keratinocyte activation) OR (dendritic cell activation) OR DPRA OR ADRA OR kDPRA OR (ARE‐Nrf2 luciferase KeratinoSens) OR (ARE‐Nrf2 luciferase LuSens) OR h‐CLAT OR U‐Sens OR (IL‐8 Luc assay) OR GARD OR EpiSensA OR (RHE‐IL18) OR SensCeeTox) was performed. We included all studies using OECD‐validated or scientifically accepted methods. The literature search to review the clinical results (contact allergy and allergic contact dermatitis) was performed using the following keywords: ((BPO) OR CAS 94‐36‐0) AND (allerg* OR dermat* OR sensitiz* OR sensitis* OR skin OR contact OR hypersensitiv* OR hypersensitiz* OR hypersensitis*). Here, we restricted the analysis to January 1999–December 2022, as data from earlier patch test studies were already summarised [20]. In total, the initial search in Pubmed retrieved 918 articles and 1082 articles were found in Web of Science. Study eligibility was first assessed based on the title and abstract. The second assessment of eligibility was based on the full text of the articles and was done by two independent investigators.

With respect to clinical data, we restricted the analysis to studies using a maximum 1% BPO in pet. for patch testing based on reported irritancy at higher concentrations [21, 22]. Application of these defined criteria resulted in 68 publications. In addition, we reviewed relevant publications that were already reviewed [20]. References in the articles obtained were also screened to identify other potential sources of information.

Individual test results of the different non‐animal methods were analysed by applying the Defined Approaches ‘2 out of 3’ and Integrated Testing Strategy version 1 (ITSv1) and 2 (ITSv2) for skin sensitisation hazard as described in OECD guideline no. 497 [15]. In detail, in the ‘2 out of 3’ approach results from DPRA, KeratinoSens and h‐CLAT are considered, and any two of the three methods determine the final outcome, that is, if two methods result positive, the overall results yield the prediction of a test chemical to be a sensitizer, while any two negative test results would yield the prediction of a test chemical to be a non‐sensitizer. In ITSv1 and ITSv2, scores are assigned for relative test results of DPRA (0–3 scores), h‐CLAT (0–3 scores) and for in silico results from the Deductive Estimation of Risk from Existing Knowledge (DEREK) Nexus (ITSv1, 0–1 scores) or the OECD QSAR Toolbox (ITSv2, 0–1 scores) to yield an overall result that predicts the skin sensitisation potency of a chemical: United Nations Globally Harmonised System (UN GHS) of Classification and Labelling of Chemicals category 1A (6–7 scores, strong sensitizer), category 1B (2–5 scores, other sensitizer), not classified (0–1 scores, non‐sensitizer) or inconclusive.

## 3 Results of the Literature Search and Data Analysis

### 3.1 Skin Sensitisation Potential and Potency of BPO

BPO was tested positive in different experimental animal studies such as the Guinea Pig Maximisation Test, Bühler Test and LLNA [8, 9, 10]. The LLNA allows also the determination of a chemicals' relative sensitisation potency based on estimation of the concentration inducing positivity (effective concentration, EC3 value), and comprehensive comparison with human data [23]. With respect to potency, BPO gave different EC3 values ranging between 0.0044% and 0.3% [10, 24, 25, 26]. This finding led to the classification of BPO as an extreme or strong sensitizer following the criteria defined by the European Centre for Ecotoxicology and Toxicology of Chemicals (ECETOC) [27].

**FIGURE 2:**
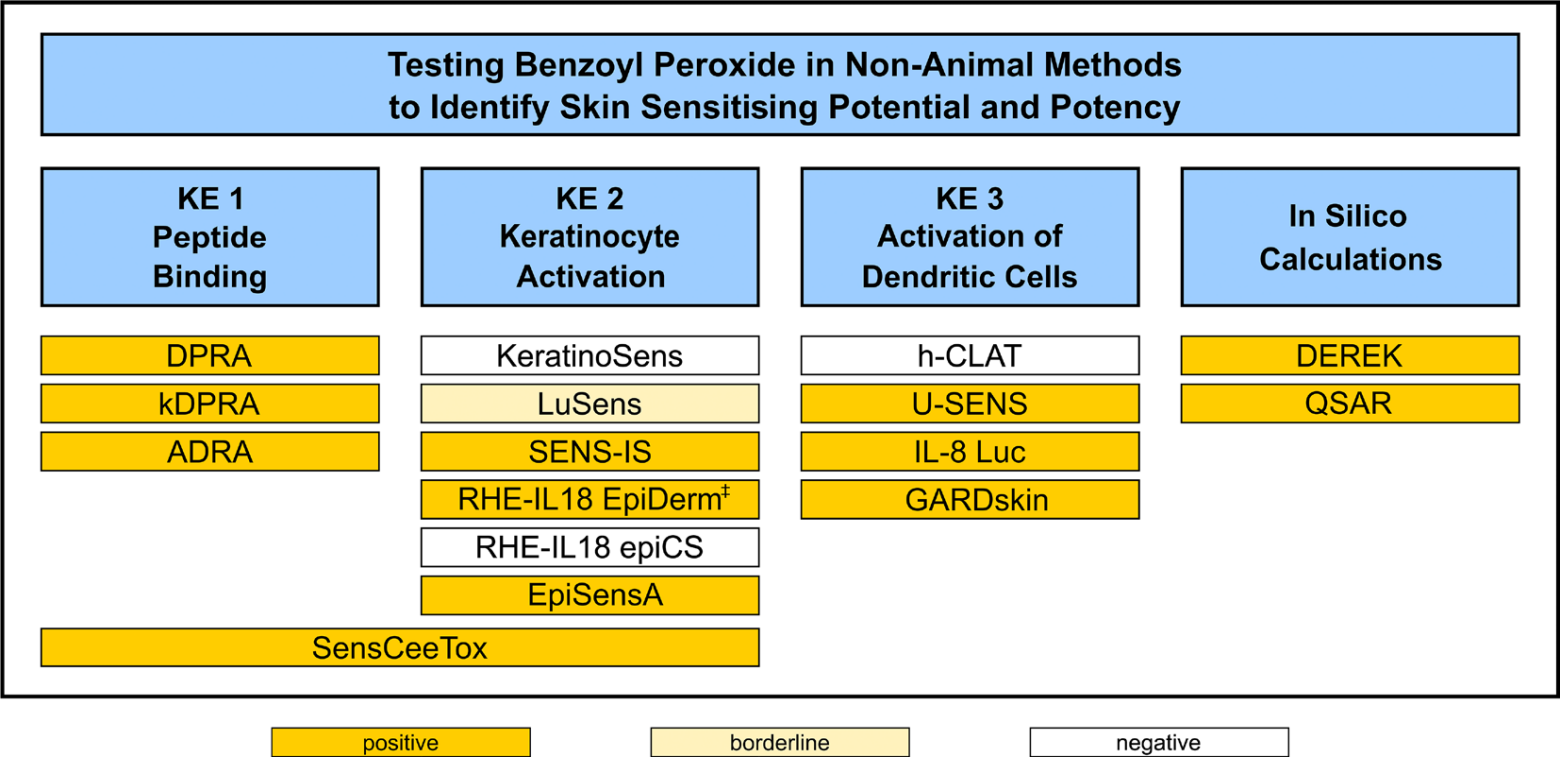
Graphical summary of testing (positive, negative, borderline (close to cut off)) BPO in non‐animal methods for skin sensitisation in KE 1 (4 methods), KE 2 (7 methods), KE 3 (4 methods) and in silico calculations (2 methods). In single methods testing KE 1, KE 2 and KE 3 as well as in silico calculations, positivity was achieved. ADRA, Amino acid Derivative Reactivity Assay [28]; DEREK, Deductive Estimation of Risk from Existing Knowledge [29]; DPRA, Direct Peptide Reactivity Assay [29]; EpiSensA, Epidermal Sensitization Assay [30]; GARDskin, Genomic Allergen Rapid Detection for assessment of skin sensitizers [31]; h‐CLAT, Human Cell Line Activation Test [29]; IL‐8 Luc, Interleukin‐8 Luciferase Assay [32]; kDPRA, Kinetic Direct Peptide Reactivity Assay [33]; KeratinoSens, Keratinocyte Sensitization Assay [29]; LuSens, Luciferase Sensitization Assay [34]; OECD QSAR, Quantitative Structure–Activity Relationship Toolbox v.3.2 [29]; RHE‐IL18 epiCs, Reconstructed Human Epidermis (EpiCS) Interleukin‐18 [35]; RHE‐IL18 EpiDerm, Reconstructed Human Epidermis (EpiDERM) Interleukin‐18 [35]; SensCeeTox, Sensitization CeeTox Inc. [35]; SENS‐IS, Testing of keratinocyte activation via analysis of the gene expression profile using a skin model [35, 36]; U‐Sens, U937 Sensitization Assay [37, 38, 39]. ^‡^ only positive with an adapted protocol.

BPO was frequently tested in non‐animal methods under development to identify the skin sensitisation potential of chemicals and possibly derive continuous potency values. Methods cover the molecular initiation event (protein reactivity, Key Event (KE) 1), activation of keratinocytes (KE 2) and dendritic cells (KE 3) and in silico calculations. BPO was tested positive in different methods addressing KE 1 (4 of 4), KE 2 (4 of 7), KE 3 (3 of 4) and in silico methods (2 of 2) (Figure 2). We evaluated the individual results by applying the Defined Approaches ‘2 out of 3’ and Integrated Testing Strategy version 1 (ITSv1) and 2 (ITSv2) (see Methods). In the ‘2 out of 3’ approach, BPO yields the prediction to be a non‐sensitizer.

In the ITSv1 and ITSv2, BPO achieved 4 of a total of 7 score points, predicting category 1B skin sensitizer according to the criteria of the Globally Harmonised System of Classification and Labelling of Chemicals.

### 3.2 Prevalence of BPO Contact Allergy in Patients With and Without Occupational Contact Dermatitis

Next, we reviewed published studies on the prevalence of contact allergy under different exposure scenarios, although (exact) exposure measurements are not available. In general, no routine patch testing of BPO in consecutive patients is performed in Europe or North America. As skin contact and sensitisation may occur because of occupational and non‐occupational exposure, the German Contact Dermatitis Research Group (DKG) for instance recommends BPO patch testing as part of several test series, namely ‘Bone Cement’, ‘Synthetic Resins and Adhesives’ and the ‘Dental Technician’. For some years, the chemical was also part of the ‘Topical Drug’, ‘Rubber’, ‘Metal Working’ and ‘Plastic and Glues’ series. Because of low clinical relevance, BPO was taken out [40, 41].

In the past, BPO (< 20%) was used to treat leg ulcers in chronic venous insufficiency and cases of Allergic Contact Dermatitis (ACD) have been reported. A considerable number of positive patch test reactions was reported in two smaller studies, namely 31 of 41 [42] and 12 of 120 (10%) [43]. In contrast, evaluation of a larger patient group found markedly lower numbers of positive patch test reactions, namely in 30 of 739 patients (3.8%) [17]. For acne patients, larger studies performed earlier than 1999 found between 0% and 1% positive reactions [17, 44]. These results contrast with only one newer small patch test study testing BPO in relation to acne therapeutics, finding 17 of 20 patients showing a positive reaction [45]. In addition, there are 14 recently published case reports with positive patch test results [46, 47, 48, 49, 50, 51, 52]. Taking into consideration the high daily BPO exposure over months of acne treatment, sensitisation to BPO seems to be a rare adverse event in acne treatment. This finding may be because of relatively low penetration, namely 3% of all BPO in a 10% formulation applied on ex vivo skin were absorbed within 8 h and the hydrolysis to benzoic acid/benzoate already in the stratum corneum may also be involved [53, 54]. Higher prevalences of up to 13% in studies involving between 193 and 311 patients with skin or mucous membrane symptoms following bone and dental implants were reported [3, 55, 56, 57, 58, 59, 60, 61]. On the other hand, prevalences around 9%–10% were reported in studies including between 126 and 539 patients with suspected allergic contact dermatitis in the context of occupational and non‐occupational handling of plastics and adhesives [62, 63, 64, 65, 66].

Variable prevalences were also seen in studies focusing on patients with occupational contact dermatitis (OCD). Table 1 provides an overview of patch test results of patients with OCD and without OCD. Twenty‐six out of 241 patients with OCD showed a positive response to BPO (10.8%), while a prevalence of 5.6% was recorded in patients without OCD. Subsequent studies investigating an association of a positive reaction to BPO among patients with OCD and professions without and with BPO handling found heterogeneous results. Table 2 shows results for mechanics and metal workers with OCD that have no occupational contact with BPO. In these groups, prevalences between 3.8% and 10.7% were found in three studies, although a certain degree of overlap has to be taken into account. Today's occupations that might be at risk of developing contact allergy to BPO are shown in Table 3. This table provides a compilation of patch test results of patients with OCD and with possible occupational exposure to BPO including larger studies and case reports. Similar to mechanics and metalworkers, prevalences between 8.7% and 10.1% were found among dental technicians that are known to have occupational contact with BPO. Surprisingly low levels of positive reactions to BPO were found for dental technicians and related professions because of possible exposure to BPO during the production of dentures, specifically for initiation of the acrylate and methacrylate polymerisation reaction [4]. However, individual cases of allergic contact dermatitis of the fingertips have been reported for dental technicians, dentists and dental assistants. Further professions that require the use of BPO when manufacturing materials may have repeated skin contact with BPO, especially during work steps where gloves cannot be worn [20, 67]. Accordingly, some positive reactions were described from carpenters, cabinet makers, model makers and technicians; electrical and electronic equipment mechanics and fitters; as well as health care professionals. Again, no clear differences between different occupational groups were apparent.

**TABLE 1:** Patch test results to BPO (1% pet.) in groups without and with OCD.

Patch test with BPO (1% pet., reading ≥ D3)	*N* (IR, ? +, pos, neg)	Sum (pos, neg)	Positive reactions (% of *N*)	References
Patients without and with OCD	29 758	26 869	2316 (7.8%)	[22]^a^
Patients without OCD	15 771	—	879 (5.6%)	[68]^a^
Patients with OCD	241	—	26 (10.8%)	[62]^a^

*Note*: Total *N*, the sum of positive and negative reactions and the number of positive reactions are shown.

Abbreviations: — no data available; ?+, doubtful reaction; IR, irritant reaction; neg, negative reaction; OCD, Occupational Contact Dermatitis; pos, positive reaction.

^a^ Overlap of patient samples possible.

**TABLE 2:** Patch test results to BPO (1% pet.) in mechanics and metal workers with OCD.

Patch test with BPO (1% pet., reading ≥ D3)	*N* (IR, ? +, pos, neg)	sum (pos, neg)	Positive reactions (% of *N*, *N* > 100)	References
Mechanics	704	—	27 (3.8%)	[69]^a^
Cutting metal workers	118	—	7 (5.9%)	
Other metal workers	103	—	11 (10.7%)	
Mechanics and metal workers	—	598	26 (n.d.)	[68]^a^
	57^b^	—	2 (n.d.)	[70]

*Note*: Total *N*, the sum of positive and negative reactions and the number of positive reactions are shown.

Abbreviations: — no data available; ?+, doubtful reaction; IR, irritant reaction; n.d., not defined; neg, negative reaction; pos, positive reaction.

^a^ Overlap of patient samples possible.

^b^ Reading day not indicated.

**TABLE 3:** Patch test results to BPO (1% pet.) in patients with OCD in different professions with contact to BPO.

Patch test with BPO (1% pet., reading ≥ D3)	*N* (IR, ? +, pos, neg)	Sum (pos, neg)	Positive reactions (% of *N*, *N* > 100)	References
Dental technicians	576	512	58 (10.1%)	[22]^a^
	199	184	19 (9.5%)	[21]^a^
	126	—	11 (8.7%)	[64]
	—	123	11 (n.d.)	[68]^a^
Dental assistants (incl. dentists)	—	180	25 (n.d.)	[68]
Dentists	79	—	2 (n.d.)	[63]
Orthopaedic technician^b^	1	1	1 (n.d.)	[71]
Electrical‐, electronic equipment mechanics and fitters	—	117	9 (n.d.)	[68]
Electrical technician	1	1	1 (n.d.)	[72]
Carpenter, cabinet maker, model maker	—	135	11 (n.d.)	[68]
Bakers	—	80	3 (n.d.)	[68]
	1	1	1 (n.d.)	[73]
Marble grinder	1	1	1 (n.d.)	[74]
Health care professionals	—	95	10 (n.d.)	[68]

*Note*: Total *N*, the sum of positive and negative reactions and the number of positive reactions are shown.

Abbreviations: — no data available; ?+, doubtful reaction; IR, irritant reaction; n.d., not defined; neg, negative reaction; pos, positive reaction.

^a^ Overlap of patient samples possible.

^b^ Reading day not indicated.

## 4 Discussion

The present article reviews available data on the sensitisation potential and potency of BPO in humans. The capacity of BPO to induce skin sensitisation has been demonstrated in experimental animal studies by evaluation of induction or elicitation. Thus, the prediction of a test chemical to be a sensitizer is derived from a single test method. In contrast, using non‐animal methods this prediction is derived from so‐called Defined Approaches that define how individual test results of a chemical have to be combined to come to the final outcome. As each method has limitations, different methods have been developed for defined KEs during the acquisition of skin sensitisation.

For BPO, negative results have been achieved with several non‐animal methods, namely KeratinoSens, h‐CLAT and RHE‐IL18 EpiCS (Figure 2). In principle, several of those methods including KeratinoSens and h‐CLAT are based on aqueous systems that may lead to false negative results and underprediction of the sensitising potential of fast hydrolysing substances. The latter is of high importance for BPO that can hydrolyse under aqueous milieu to benzoic acid/benzoate which is a very weak sensitizer. In line, benzoic acid tested negative in various non‐animal methods, including DPRA, KeratinoSens and h‐CLAT [29]. On the other hand, BPO has also been tested positive in methods assessing peptide reactivity (KE 1), even under aqueous conditions. This apparent contradiction could be based on the fact that these methods are carried out with comparably higher concentrations compared with KeratinoSens and h‐CLAT and thus assure that sufficient non‐hydrolysed BPO is available for reaction. Testing BPO with non‐aqueous methods based on skin models, positive results were obtained with two out of three methods. Furthermore, not all methods are able to detect all classes of chemical reactivity of known sensitizers. For instance, acylating agents are tested negative with KeratinoSens [13]. As BPO acts as an acylating agent, the negative test result in this assay seems plausible. Thus, applying the Defined Aapproaches according to OECD [15] leads to heterogeneous final outcomes. Because of the negative result with the KeratinoSens, for BPO, the prediction is to be a non‐sensitizer in the ‘2 out of 3’ approach. However, it should be clearly acknowledged that the ‘2 out of 3’ approach was shown to have a high accuracy (89%) in predicting human skin sensitisation hazard and its accuracy is comparable to the predicted accuracy of the LLNA (82%) [15]. Furthermore, the Ddefined Approaches are contiguously improved [75, 76]. For instance, considering the positive result of BPO in U‐SENS instead of the negative h‐CLAT, the final outcome is positive.

Furthermore, the second type of integration strategies, namely Integrated Testing Strategies version 1 (ITSv1) and 2 (ITSv2) do not consider the results of the KeratinoSens. Both strategies allow one to decide whether a substance is a significant skin sensitizer and, if so, whether it is a stronger (category 1A) or weaker (category 1B) skin sensitizer according to the criteria of the United Nations Globally Harmonised System (UN GHS) of Classification and Labelling of Chemicals. It should be noted that a lower category ‘not classified’ exists containing both true non‐sensitizers and the very weakest skin sensitizers. These GHS categories based on animal testing can be mapped to human potency categories [77]. For instance, a substance categorised into 1B has a low to moderate frequency of contact allergy and/or a low to moderate potency based on its evaluation in the murine LLNA, in which potency is quantified by the EC3 value. In the latter assay, BPO has been classified as an extreme to strong sensitizer. In contrast, ITSv1 and ITSv2 predicted BPO correctly but categorised BPO as a weaker skin sensitizer (category 1B). There is a clear disparity between its relative skin‐sensitising potency in the well‐established predictive LLNA test versus ITSv1 and ITSv2. The discrepancy is also apparent when the frequency with which BPO induces contact allergy is taken into account. Indeed, it remains unresolved why only very few positive patch test reactions were reported among patients with extensive BPO exposure during acne treatment. We found only a few cases in the more recent literature, while a prevalence of 1% was reported earlier [44]. Thus, the potency prediction of BPO using non‐animal methods (ITSv1 and ITSv2) reflects the human situation better than the prediction based on the LLNA. It can be speculated that the higher potency of BPO observed in mice could be explained by the thinner stratum corneum of mice, which reduces BPO hydrolysis to the very weak and unclassified skin sensitizer benzoic acid/benzoate, which has already been detected in this part of the epidermis [78]. The different potency of BPO and benzoic acid/benzoate is further supported by patch test studies indicating that only 2.7% of the cases that reacted positive to BPO (1% pet.) react to sodium benzoate (5% pet.) [79].

Overall, positive reactions to BPO and high prevalences (up to 10%) were observed among groups with and without occupational exposure. In general, a positive patch test may be because of the molecule itself or because of sensitisation to another, structurally related chemical [80]. Indeed, cross‐elicitation by similar molecules most likely because of T‐cell receptor cross‐reactivity caused by allergen‐specific T lymphocytes has been observed for certain chemicals [81]. Specifically BPO can be degraded to benzoic acid/benzoate within the stratum corneum. Consequently, dermal application of BPO may elicit positive reactions in persons with sensitisation to benzoic acid/sodium benzoate, which is a widely used substance and cosmetic ingredient but a very weak allergen. Regardless, a + positive patch test response to BPO must be interpreted cautiously, as this concentration can trigger false‐positive patch test reactions (0.6%–2.3%) [1, 82]. Therefore, we and others did not consider studies using higher patch test concentrations [22, 83]. Distinction between irritant and allergic patch test responses to BPO—as with any allergen—is crucial [21, 83]. Consequently, several readings are needed to discriminate a one plus positive (+) decrescendo reaction from allergic reactions, and it is debatable whether a single + patch test reaction to BPO at D2 is truly an indicator of weak sensitisation. Furthermore, there are several studies that draw attention to a lack of clinical relevance of + reactions compared with ++ and +++ reactions [20, 22]. Nevertheless, only a few studies provide results on several reading days and the strength of the reactions. Table 4 shows a compilation of studies providing patch test scores to BPO (?+; +; ++; +++). Overall, few ++ and +++ reactions were observed, varying between 0.6% and 2.5% of tested patients. Again, no significant increase in ++ and +++ patch test responses was observed among acne patients. The same was observed testing dental technicians with hand eczema and likely BPO exposure. The integration of BPO in a baseline series is indeed not justified, taking into account the above‐mentioned difficulties and the low probability of finding relevant responses in unselected patients. When BPO patch testing is indicated, readings should be performed on multiple days. In cases with occupational exposure, testing with several dilutions might be considered. Regardless of the context, the clinical relevance of any positive result must be thoroughly assessed to ensure its significance in the individual's exposure history. In certain cases, the Safety Data Sheet (SDS) of BPO‐containing products may provide information on the amounts of BPO. If the reaction is caused by residues, it will be more difficult to identify them, as they are usually not declared in the SDS. However, several simple analytical methods such as colorimetry, fluorescence [84] or high‐performance liquid chromatography [85] have been developed to detect BPO.

**TABLE 4:** Scoring of patch test responses to BPO (1% pet.) in patients with and without OCD.

Patch test with BPO (1% pet., reading ≥ D3)	Irritant reaction	Scoring according to reference				References
		?+	+	++	+++	
Patients with and without OCD (%)						
29 758	619 (2.1%)	2270 (7.6%)	1931 (6.5%)	326 (1.1%)	59 (0.2%)	[22]^a^
3758^b^	86 (2.3%)	318 (8.5%)	235 (6.3%)	45 (1.2%)	14 (0.4%)	[79]
721	4 (0.6%)	57 (7.9%)	33 (4.6%)	2 (0.3%)	2 (0.3%)	[67]
Patients with OCD (%)						
Dental technicians 199	4 (2%)	11 (5.5%)	14 (7.0%)	3 (1.5%)	2 (1%)	[21]^a^
Dental technicians 41^c^	0	10	2	2	0	[67]
Metal workers 1128	8 (0.7%)	99 (8.8%)	49 (4.3%)	6 (0.5%)	2 (0.2%)	[41]
Patients without OCD						
Acne 20^c^	3 (?+/IR)	—	14	3	0	[45]
Acne 7^c^	0	0	2	4	1	[46]
Ulcus cruris 20^c^, ^d^ (partly testing with 0.5%)	—	—	4	2	3	[86]

*Note*: The number of irritant, doubtful, +, ++ and +++ reactions are indicated.

Abbreviations: — no data available; ?+ doubtful reaction; IR, irritant reaction; OCD, occupational contact dermatitis.

^a^ Overlap of patient samples possible.

^b^ Reading day not indicated.

^c^ No % calculated for *N* < 100.

^d^ Partly testing with 0.5% BPO.

## 5 Conclusion

Experimental animal studies and non‐animal methods demonstrate the skin sensitising potential of BPO. Contact allergy and ACD to BPO have been documented in patients without and with OCD, indicating both non‐occupational and occupationally acquired sensitisation. As there are no clear differences in prevalences between occupational groups with and without exposure, no reliable evidence of an increased risk of sensitisation because of occupational exposure in the dental sector or of electromechanical and adhesive processing workers was found.

## Author Contributions

**Kerstin Egele:** writing – original draft, investigation, methodology, writing – review and editing, visualization, data curation. **Hans Drexler:** writing – review and editing, methodology. **Manigé Fartasch:** writing – review and editing, methodology. **Vera van Kampen:** writing – review and editing, methodology. **Hans F. Merk:** writing – review and editing, methodology. **Dennis Nowak:** writing – review and editing, methodology. **Axel Schnuch:** writing – review and editing, methodology. **Wolfgang Uter:** writing – review and editing, methodology. **Patricia Kreis:** writing – review and editing. **Brunhilde Blömeke:** funding acquisition, writing – original draft, methodology, writing – review and editing, project administration, supervision.

## Conflicts of Interest

The authors declare no conflicts of interest [Correction added on 25 March 2025, after first online publication: Conflict of interest was inadvertently removed and has been reinstated in this version.].

## Data Availability

Data sharing is not applicable to this article as no new data were created or analyzed in this study.
